# Comparative thermal research on tetraazapentalene-derived heat-resistant energetic structures

**DOI:** 10.1038/s41598-020-78980-1

**Published:** 2020-12-10

**Authors:** Jing Zhou, Li Ding, Yong Zhu, Bozhou Wang, Xiangzhi Li, Junlin Zhang

**Affiliations:** 1 State Key Laboratory of Fluorine and Nitrogen Chemical, Xi’an Modern Chemistry Research Institute, Xi’an, 710065 China; 2 Department of Chemistry, Technische Universität München, 85748 Garching Bei München, Germany

**Keywords:** Chemistry, Materials chemistry

## Abstract

Organic inner salt structures are ideal backbones for heat-resistant energetic materials and systematic studies towards the thermal properties of energetic organic inner salt structures are crucial to their applications. Herein, we report a comparative thermal research of two energetic organic inner salts with different tetraazapentalene backbones. Detailed thermal decomposition behaviors and kinetics were investigated through differential scanning calorimetry and thermogravimetric analysis (DSC-TG) methods, showing that the thermal stability of the inner salts is higher than most of the traditional heat-resistant energetic materials. Further studies towards the thermal decomposition mechanism were carried out through condensed-phase thermolysis/Fourier-transform infrared (in-situ FTIR) spectroscopy and the combination of differential scanning calorimetry-thermogravimetry-mass spectrometry-Fourier-transform infrared spectroscopy (DSC-TG-MS-FTIR) techniques. The experiment and calculation results prove that the arrangement of the inner salt backbones has great influence on the thermal decompositions of the corresponding energetic materials. The weak N4-N5 bond in “y-” pattern tetraazapentalene backbone lead to early decomposition process and the “z-” pattern tetraazapentalene backbone exhibits more concentrated decomposition behaviors.

## Introduction

**Figure 1:**
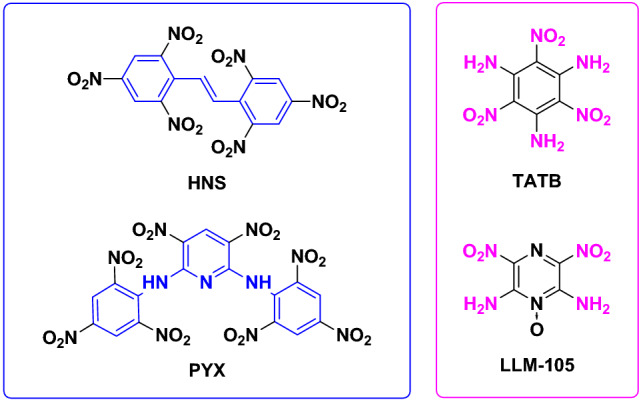
Representative heat-resistant energetic materials.

Research on heat-resistant energetic materials has been intensively conducted worldwide during the past decades. In practice, the improvement of thermal stability is crucial to explosives and propellants working under high temperatures^1–3^. From structural point of view, expansion of the aromatic structures and introduction of amino groups are currently the most efficient strategies to improve the thermal stabilities of energetic materials^4,5^. Such effects are attributed to the additional stabilization effect achieved from the strengthened conjugation and hydrogen bond systems^6–9^. Based on these strategies, a series of energetic structures, such as 2,2′,4,4′,6,6′-hexanitrostilbene (HNS)^10^, 2,6-bis(picrylamino)-3,5-dinitropyridine (PYX)^11^, 1,3,5-triamino-2,4,6-trinitrobenzene (TATB)^12^, and 2,6-diamino-3,5-dinitropyrazine-1-oxide (LLM-105)^13^, have been successfully synthesized and endowed with good thermal stabilities (Fig. 1). However, the number of heat-resistant energetic materials is still highly limited that is difficult to meet the increasingly demanding application requirements.

**Figure 2:**
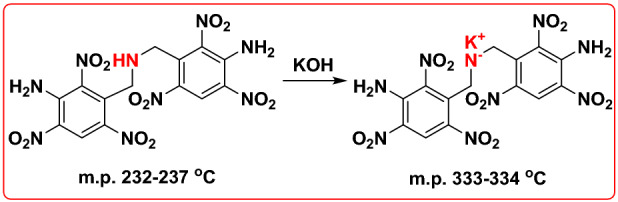
Thermal stability improved by salt formation.

Salt formation is believed as an alternative approach to improve the thermal stability of energetic materials and this can be illustrated by the comparison of the melting points between 3,3′-diamino-2,2′,4,4′,6,6′-hexanitrodiphenylamine and its potassium salt^14^ (Fig. 2). However, most salts suffer from severe hygroscopicity which hinders the application of salt formation strategy in heat-resistant energetic materials^15,16^. Different from traditional salt structures, most organic inner salt structures successfully avoid the problems caused by hygroscopicity, making them potential ideal backbones for the synthesis of heat-resistant energetic materials. Despite all these, detailed and systematic research towards the thermal behaviors and pyrolysis mechanism of energetic organic inner salt structures are rarely reported.

**Figure 3:**
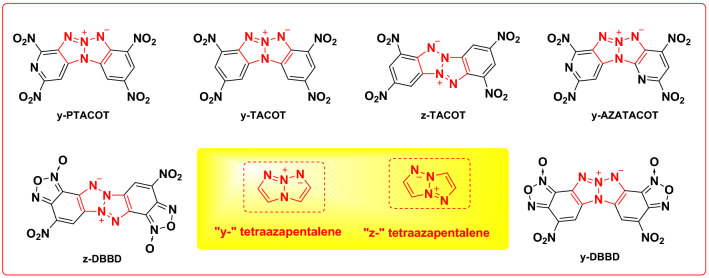
Energetic materials based on “y-” and “z-” tetraazapentalene backbones.

Tetraazapentalenes are novel inner salt structures which are ideal for the design of energetic materials and their core nitrogen atoms can be arranged in both “y-” and “z-” patterns^17^. The incorporation of nitro groups into “y-” and “z-” tetraazapentalene backbones have been explored in attempt to synthesize energetic structures (TACOT) with impressive thermal stabilities^18–21^. Further nucleophilic displacement of the *o*-nitro groups in TACOT with azide anion followed by nitration and heating will lead to the formations of similar energetic structures of DBBD^22,23^ (Fig. 3).

**Figure 4:**
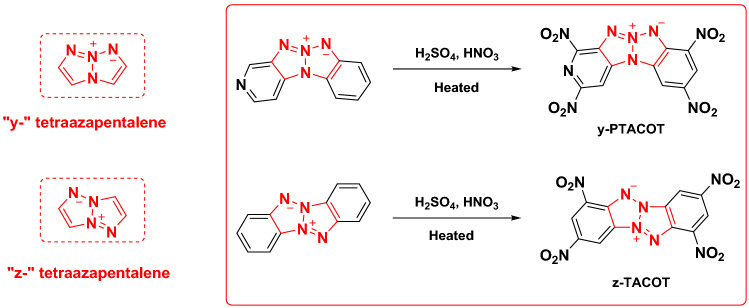
y-PTACOT and z-TACOT structures.

Herein, we focused on a comparative thermal research of two energetic organic inner salts, y-PTACOT and z-TACOT, which were prepared based on “y-” and “z-” tetraazapentalene backbones (Fig. 4). Both experiment and calculation methods are applied to clarify the stabilization effect achieved from the inner salt backbones and provide further clues to elucidate the pyrolysis mechanisms during the heating process. The comparative studies can also improve the understanding on how the arrangement of the inner salt backbones and elemental composition in the aromatic rings affect their thermal behaviors.

## Results and discussion

**Figure 5:**
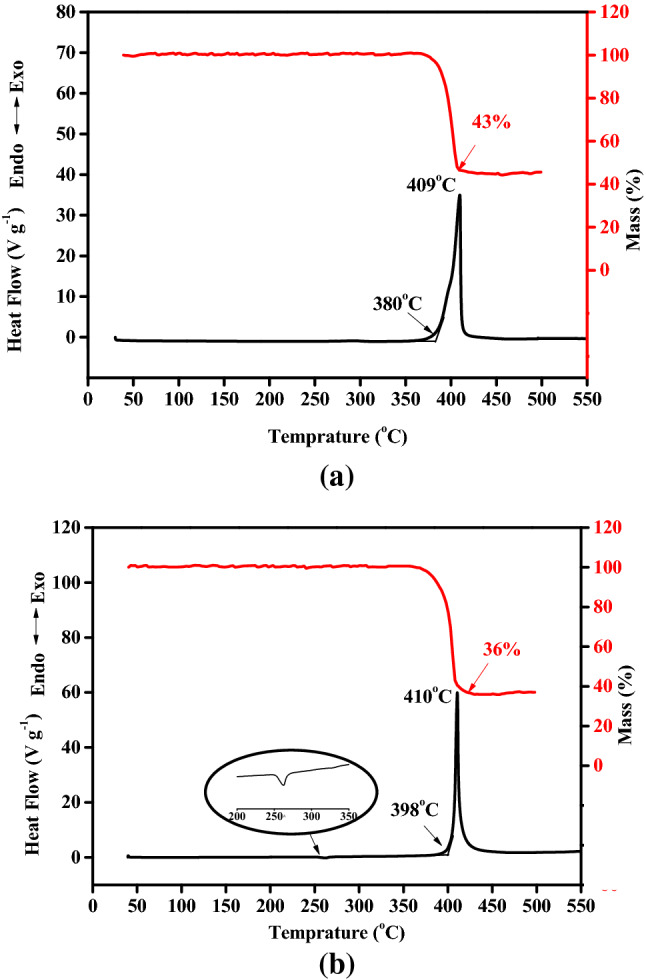
DSC-TG curves of y-PTACOT (**a**) and z-TACOT (**b**).

Both the backbones of y-PTACOT and z-TACOT are based on tetraazapentalene core structures but arranged in different patterns. As potential heat-resistant energetic materials, the detailed thermal decomposition behaviors were first investigated and compared. As shown in Fig. 5, y-PTACOT and z-TACOT exhibited similar thermal decomposition processes in the range of 0–500 °C. The DSC curve of y-PTACOT and z-TACOT at a heating rate of 10 °C min^−1^ was consisted of single exothermic decomposition peaks at 409 °C and 410 °C, respectively, which were much higher than those of most traditional energetic materials. The thermal decompositions of y-PTACOT and z-TACOT were speculated to be one-step reactions with close weight loss data about 64% and 57%, respectively. However, the heat releasing process of z-TACOT was more concentrated than that of y-PTACOT.

**Figure 6:**
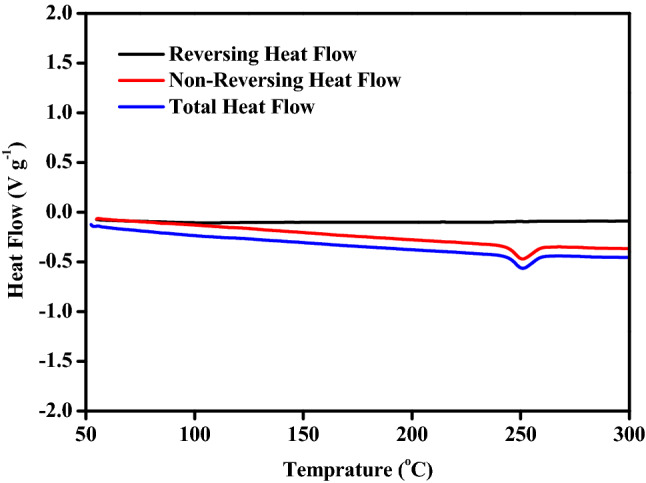
MDSC measurements of z-TACOT.

It is noteworthy that a week endothermic peak was observed at about 260 °C during the decomposition process of z-TACOT with no weight loss, indicating a physical change like melt or crystal transformation. To further investigate this physical change, modulated DSC (MDSC) measurements^24^ of z-TACOT were presented. The endothermic peak was only found in the non-reversing heat flows, which means this physical change is irreversible and crystal transformation is the maximum possible process (Fig. 6).

**Figure 7:**
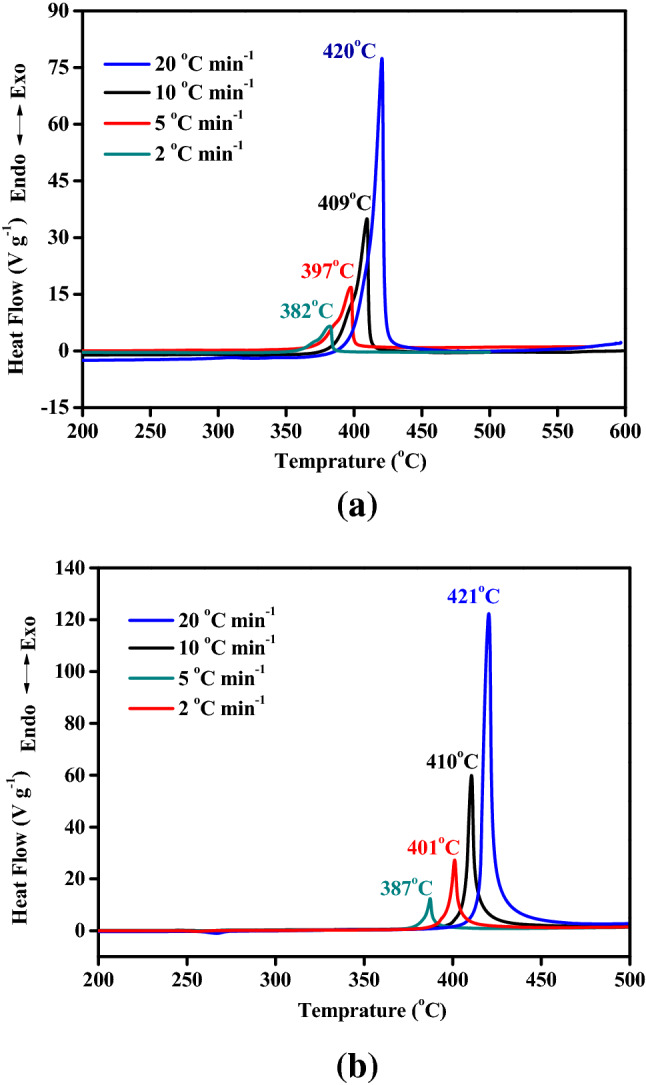
DSC measurements of y-PTACOT (**a**) and z-TACOT (**b**) at the heating rates of 2, 5, 10 and 20 °C min^−1^.

Non-isothermal kinetic studies on thermal decompositions of y-PTACOT and z-TACOT were carried out based on DSC experiments employed at different heating rates (Fig. 7). The thermal decomposition peak and heat release amount for y-PTACOT and z-TACOT at the heating rates of 2, 5, 10 and 20 °C min^−1^ were summarized in Table 1. It is clear that the decomposition peak shifted towards high temperatures with the increase of heating rate while the decomposition heat was almost unchanged, further proved that these thermal decompositions are one-step reactions.

**Table 1:** Thermal decomposition parameters of y-PTACOT and z-TACOT.

*β*^a^ (°C min^−1^)	y-PTACOT		z-TACOT	
	T_p_^b^ (K)	ΔH^c^ (J/g)	T_p_^b^ (K)	ΔH^c^ (J g^−1^)
2	655	2767	660	2372
5	670	2637	674	2394
10	682	2651	683	2337
20	693	2655	694	2369

^a^β, heating rate; ^b^T_p_, peak temperature (K); ^c^ΔH, decomposition heat.

**Figure 8:**
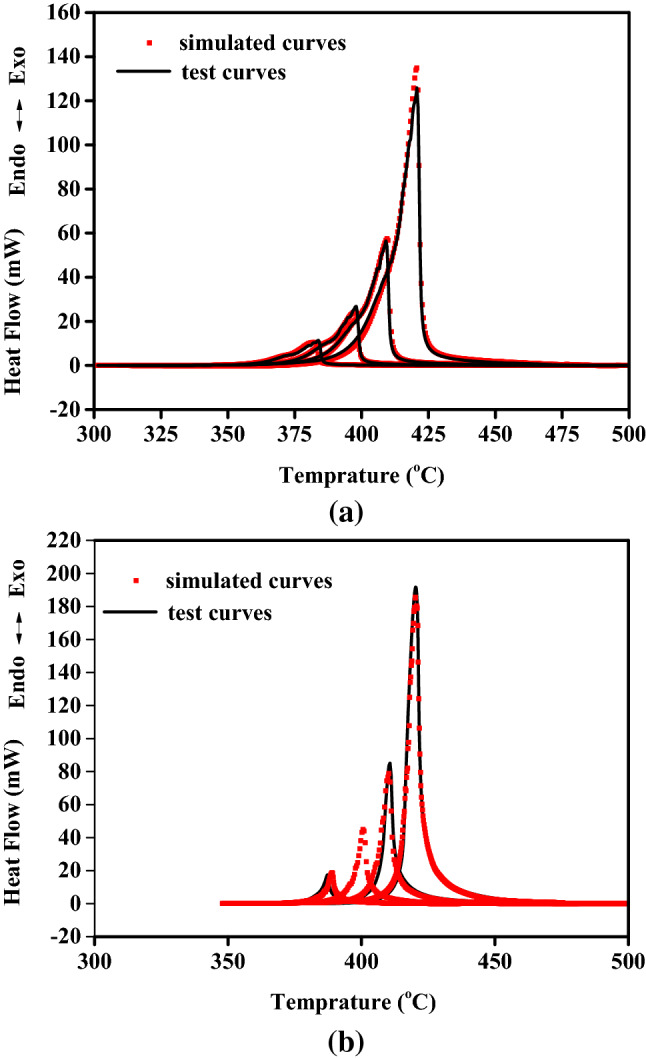
DSC measurements of y-PTACOT (**a**) and z-TACOT (**b**) at the heating rates of 2, 5, 10 and 20 °C/min.

Calculation studies of the kinetic parameters and mechanism functions of the decomposition reaction of y-PTACOT and z-TACOT were carried out with NETZSCH Thermokinetics Software. Friedman method, a model-free algorithm, was first employed to predict the types of the decomposition reactions of y-PTACOT and z-TACOT. The preliminary calculation results from Friedman method showed that both of the two decomposition reactions were acceleration processes. Meanwhile, based on the preliminary calculation results of apparent activation energies and pre-exponential constants, various reaction model algorithms were further applied for identifications of the reaction mechanisms. Multiple autocatalytic acceleration and nucleation reaction models were tested and proved that the decomposition reactions of y-PTACOT and z-TACOT agreed with nth order autocatalytic model (C_n_) and extended Prout-Tompkins model (BNA), respectively. Figure 8 showed the overlay of fitted and measured curves and the results proved that prediction curves of y-PTACOT and z-TACOT coincided with their measurement results well. The kinetic parameters were summarized in Table 2 and the apparent activation energy of z-TACOT was higher than that of y-PTACOT, which may explain the better stability of z-TACOT.

**Table 2:** Calculated thermal decomposition parameters of y-PTACOT and z-TACOT with mathematical models.

nth order autocatalytic model (C_n_)			
Activation Energy (Ea, kJ/mol)	LogA	React order n	Log(AutocatA)
y-PTACOT			
226.164	14.690	0.393	1.148

Extended Prout-Tompkins model (BNA)			
Activation Energy (Ea, kJ/mol)	LogA	Autocat order	–
z-TACOT			
262.930	19.197	1.107	–

**Figure 9:**
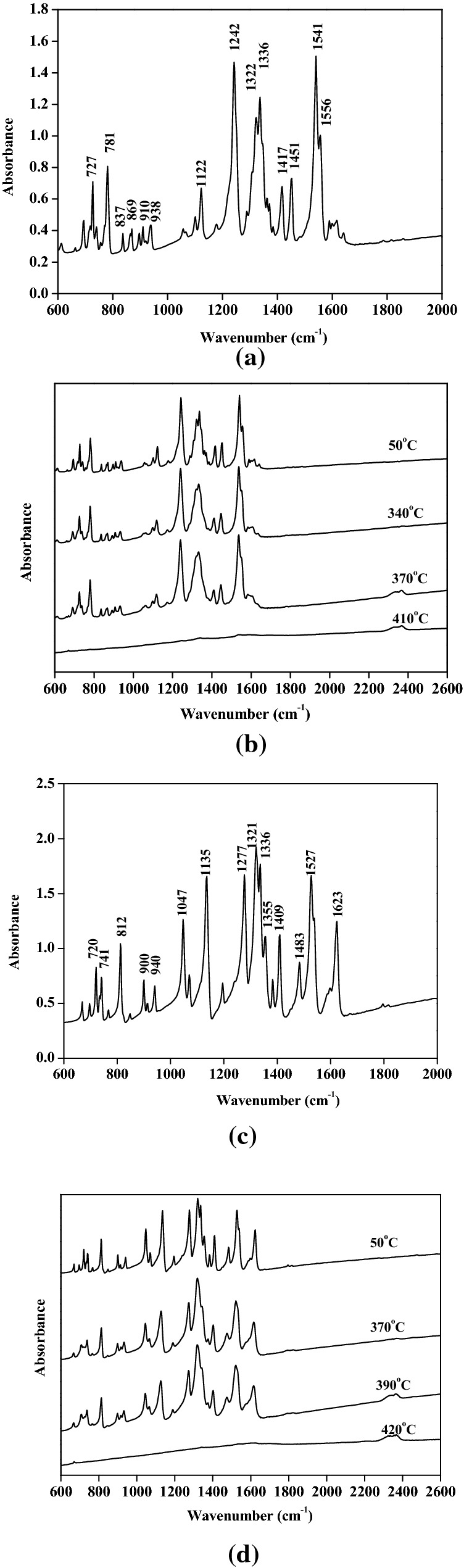
FTIR experiments of y-PTACOT and z-TACOT at room temperatures (**a** and **c**) and high temperatures (**b** and **d**).

To further study the mechanisms of the thermal decompositions of y-PTACOT and z-TACOT, the thermal decomposition of y-PTACOT and z-TACOT were then investigated through condensed-phase thermolysis/Fourier-transform infrared (in-situ FTIR) spectroscopy method and their FTIR spectra at different temperatures are shown in Fig. 9. Figure 9b showed that there was almost no structure change of y-PTACOT before 340 °C. With the further increasing the heat temperature, the signals of tetraazapentalene structure started to weaken at 370 °C and weak peaks around 2350 cm^−1^ emerged and strengthened gradually. Signals of tetraazapentalene structure disappeared completely after 410 °C. The new signals around 2350 cm^−1^ may belong to the alkynyl moieties. Compared with tetraazapentalene structure, the dinitropyridine structure in z-TACOT exhibited superior stability with much slower absorption peaks decrease rate which started from 370 °C and disappeared completely at 420 °C (Fig. 9d). Similar new signals around 2350 cm^−1^ were able to be observed at 390 °C. It is clear that both the arrangement of the inner salt backbones and the introduction of nitrogen atom have influence on the thermal decompositions of the corresponding energetic materials.

**Figure 10:**
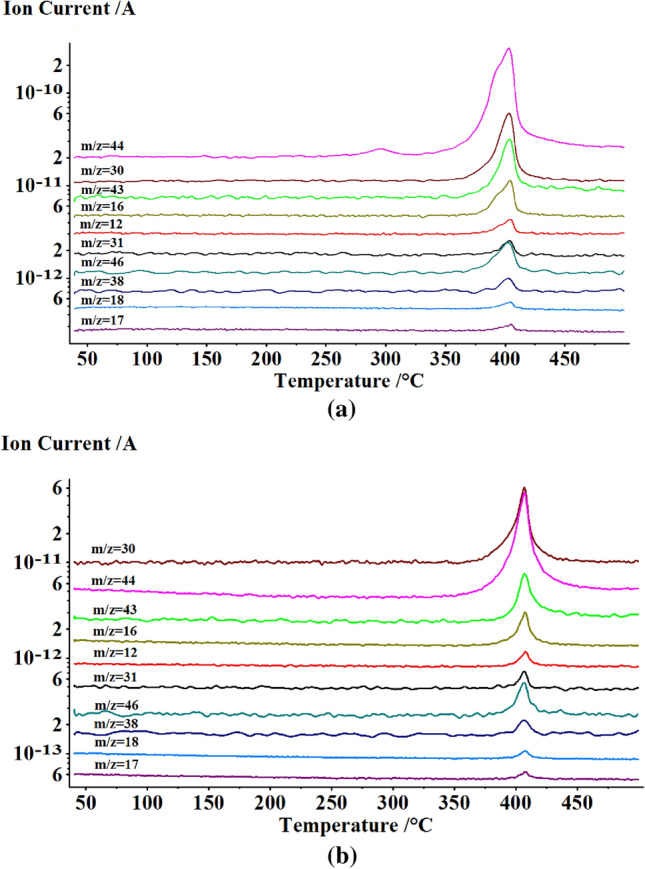
Ion current curves of gaseous products of the thermal decompositions of y-PTACOT (**a**) and z-TACOT (**b**) from simultaneous DSC-TG-FTIR-MS experiments.

Simultaneous DSC-TG-FTIR-MS experiments were also performed for the investigation of the differences in evolved gases products (Fig. 10). Ion current curves of gaseous products of the thermal decompositions of y-PTACOT and z-TACOT were shown in Fig. 10a,b, respectively. Figure 10a showed the analysis result of the mass spectrum fragments of y-PTACOT and the MS signals were found at m/z values of 17, 18, 12, 31, 38, 46, 16, 43, 30 and 44 at about 400 °C, in order of ascending intensity. From elementary composition standpoint, the major fragments at m/z values of 46, 16, 43, 30 and 44 correspond to NO_2_, NH_2_, N_3_H, NO and CO_2_-N_2_O fragments. The results were also supported by the absorption peaks from FTIR data (Figure S1). In the DSC-TG-FTIR-MS experiments, the strong signals between 2200 and 2400 cm^−1^ belonged to CO_2_ and N_2_O while the signals between 1800 and 1950 cm^−1^ belonged to NO. It was also clear that the major fragments at m/z values of 44 and 30 stated much earlier than the other fragments. Since the in-suit FTIR experiment had proved that the tetraazapentalene moieties decomposed prior to the dinitropyridine moieties, this result reflected the first appeared fragments were caused by the decomposition of “y-” tetraazapentalene structure. Interestingly, similar results were obtained by the analysis of ion current curves of gaseous products (Fig. 10b) and corresponding absorption peaks from the FTIR data in DSC-TG-FTIR-MS experiments (Figure S2) of z-TACOT, however, the fragments appeared at higher temperatures and in a much more concentrated way, which also agreed with the results from in-situ FTIR experiments (Fig. 9d).

**Figure 11:**
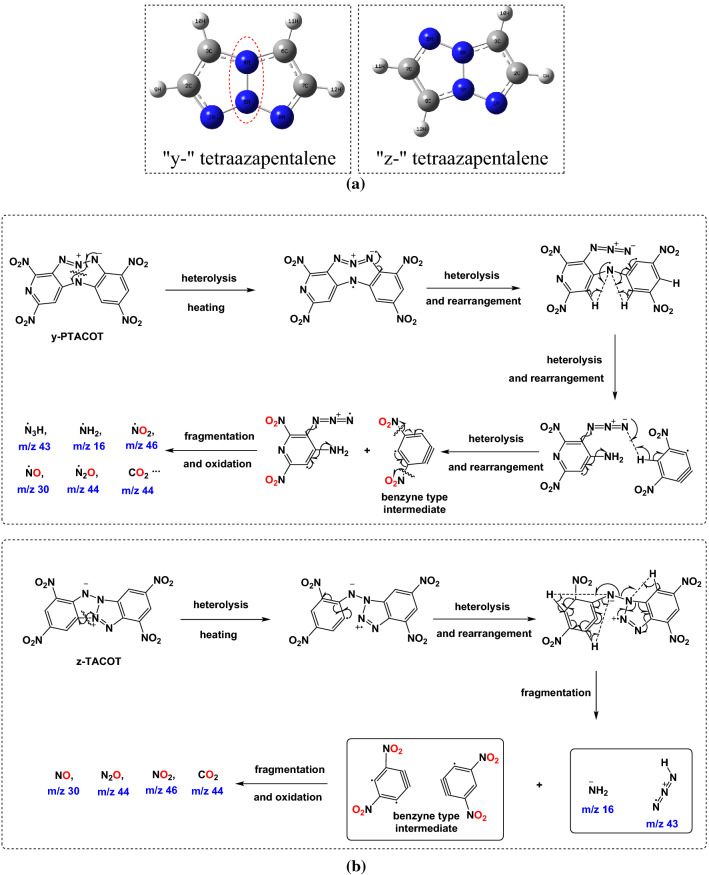
Proposed thermal decomposition mechanisms of y-PTACOT and z-TACOT.

From structural point of view, the poly-nitrogen core structures based on the poly-nitrogen core structures are thermally less stable parts in the structures of y-PTACOT and z-TACOT due to the thermal activity of poly-nitrogen species. Based on results of the in-situ FTIR and DSC-TG-FTIR-MS experiments, decomposition processes start from the tetraazapentalene moieties and the “y-” tetraazapentalene structure suffers from greater fragility. Calculation methods were carried out to check the bond orders of the “y-” and “z-” core structures, finding that the bond order of N_4_–N_5_ in “y-” tetraazapentalene structure is only about 0.85 which are much lower than other bond orders in the poly-nitrogen core structures of “y-” and “z-” poly-nitrogen core structures (Fig. 11a and Table S1, S2). The low bond order value of N_4_–N_5_ in “y-” tetraazapentalene structure indicated the weak bond stability. On the basis of the above-described analytical and calculated results, we deduced plausible thermal decomposition mechanisms as illustrated in Fig. 11b.

## Conclusions

Comparative research on the detailed thermal behaviors of y-PTACOT and z-TACOT, two energetic materials based on tetraazapentalene backbones with different arrangement, was carried out through experimental and calculation methods. The two inner salt structures exhibited similar thermal decomposition behaviors with much higher decomposition temperatures than those of most traditional energetic materials. The endothermic peak observed at about 260 °C during the decomposition process of z-TACOT was proved to be an irreversible physical change through MDSC measurements and a possible crystal transformation process was proposed. Calculation studies of the kinetic parameters and mechanism functions of the decomposition reaction of y-PTACOT and z-TACOT showed that the decomposition reactions of z-TACOT and y-PTACOT agreed with nth order autocatalytic model (C_n_) and extended Prout-Tompkins model (BNA), respectively. The apparent activation energy of z-TACOT was higher than that of y-PTACOT. Similar results were found from the DSC-TG-FTIR-MS experiments of y-PTACOT and z-TACOT, however, the fragments of z-TACOT appeared at higher temperatures and in a much more concentrated way. Compared with tetraazapentalene structure, the nitropyridine structure in y-PTACOT exhibited better stability with much slower absorption peaks decrease rate. In-suit FTIR experiment result of z-TACOT varied from that of y-PTACOT in which the signals of both tetraazapentalene and dinitrobenzene structures weakened almost synchronously. Obviously, the arrangement of the inner salt backbones has great influence on the thermal decompositions of the corresponding energetic materials.

## Methods

The samples of y-PTACOT and z-TACOT were prepared according to the reported nitration methods^20,21^. Thermal analysis experiments were performed with differential scanning calorimetry (DSC) Q250 instrument (TA, America) and model TG-DSC STA 449F3 instrument (NETZSCH, Germany). Operation conditions: sample mass, 0.5 mg; atmosphere, dynamic nitrogen; aluminum cell. IR spectra was recorded on a Nicolet 60SX FTIR spectrometer with HgCdTe detector. Condensed-phase thermolysis/Fourier-transform infrared (in-situ FTIR) spectroscopy studies were carried out with Nicolet 60 SXR FTIR spectrometer. Operation conditions: sample mass, 1.0 mg; heating rate, 10 °C min^−1^; resolution, 4 cm^−1^; spectral acquisition rate, 7.5 file min^−1^, 8 scans file^−1^; temperature range, 50–470 °C.

## Supplementary information

Supplementary Information 1.

## Data Availability

All data generated or analyzed during this study are included in this published article (and its Supplementary Information Files).
