# Supplementary material for: Comparative thermal research on tetraazapentalene-derived heat-resistant energetic structures
Source: Sci Rep. 2020 Dec 10;10:21757. doi: 10.1038/s41598-020-78980-1 (PMC7730128; doi:10.1038/s41598-020-78980-1)
Supplement: Supplementary file 1 — Supplementary Information 1. [file 41598_2020_78980_MOESM1_ESM.docx]

Comparative Thermal Research on Tetraazapentalene-Derived Heat-resistant Energetic Structures

Jing Zhou^1^, Li Ding^1^*, Yong Zhu^1^, Bozhou Wang^1^*, Xiangzhi Li^1^ and Junlin Zhang ^1,2^*

^1^ Jing Zhou, Li Ding, Yong Zhu, Bozhou Wang, Xiangzhi Li, Junlin Zhang, State Key Laboratory of Fluorine & Nitrogen Chemical, Xi’an Modern Chemistry Research Institute, Xi’an, 710065, China, E-mails: [dingli403@sina.com](mailto:dingli403@sina.com) (Li Ding); [wbz600@163.com](mailto:wbz600@163.com) (Bozhou Wang); junlin-111@163.com (Junlin Zhang)

^2^ Junlin Zhang, Department of Chemistry, Technische Universität München, Garching bei München, 85748, Germany

**Figure S1**

**Figure S2**

**Table S1**

Atom-atom overlap-weighted NAO bond order of y-PTACOT:

Atom 1 2 3 4 5 6 7 8 9

---- ------ ------ ------ ------ ------ ------ ------ ------ ------

1. N 0.0000 1.0921 -0.0274 -0.0642 0.9813 0.0097 0.0094 -0.0263 0.0049

2. C 1.0921 0.0000 1.1661 -0.0209 -0.0564 0.0075 -0.0005 0.0094 0.7785

3. C -0.0274 1.1661 0.0000 0.9505 -0.0488 -0.0071 0.0075 0.0097 0.0131

4. N -0.0642 -0.0209 0.9505 0.0000 0.8515 0.9505 -0.0209 -0.0642 0.0034

5. N 0.9813 -0.0564 -0.0488 0.8515 0.0000 -0.0488 -0.0564 0.9813 0.0058

6. C 0.0097 0.0075 -0.0071 0.9505 -0.0488 0.0000 1.1661 -0.0274 0.0000

7. C 0.0094 -0.0005 0.0075 -0.0209 -0.0564 1.1661 0.0000 1.0922 0.0000

8. N -0.0263 0.0094 0.0097 -0.0642 0.9813 -0.0274 1.0922 0.0000 0.0000

9. H 0.0049 0.7785 0.0131 0.0034 0.0058 0.0000 0.0000 0.0000 0.0000

10. H 0.0031 0.0220 0.7821 0.0060 0.0044 -0.0011 0.0000 -0.0001 -0.0011

11. H -0.0001 0.0000 -0.0011 0.0060 0.0044 0.7821 0.0220 0.0031 0.0000

12. H 0.0000 0.0000 0.0000 0.0034 0.0058 0.0131 0.7785 0.0049 0.0000

**Table S2**

Atom-atom overlap-weighted NAO bond order of z-TACOT:

Atom 1 2 3 4 5 6 7 8 9

---- ------ ------ ------ ------ ------ ------ ------ ------ ------

1. N 0.0000 1.1130 -0.0366 -0.0425 0.7423 0.0096 0.0015 -0.0372 0.0080

2. C 1.1130 0.0000 1.1391 -0.0194 -0.0495 0.0018 0.0012 0.0042 0.7811

3. C -0.0366 1.1391 0.0000 0.8913 -0.0465 -0.0376 0.0050 0.0191 0.0124

4. N -0.0425 -0.0194 0.8913 0.0000 0.9577 0.8144 -0.0579 -0.0537 0.0029

5. N 0.7423 -0.0495 -0.0465 0.9577 0.0000 -0.0476 -0.0072 0.9519 0.0038

6. N 0.0096 0.0018 -0.0376 0.8144 -0.0476 0.0000 1.0644 -0.0264 0.0000

7. C 0.0015 0.0012 0.0050 -0.0579 -0.0072 1.0644 0.0000 1.1850 0.0000

8. C -0.0372 0.0042 0.0191 -0.0537 0.9519 -0.0264 1.1850 0.0000 -0.0001

9. H 0.0080 0.7811 0.0124 0.0029 0.0038 0.0000 0.0000 -0.0001 0.0000

10. H 0.0038 0.0145 0.7896 0.0027 0.0028 0.0002 0.0000 -0.0002 -0.0002

11. H 0.0000 0.0000 0.0000 0.0039 0.0021 0.0091 0.7827 0.0175 0.0000

12. H -0.0016 0.0001 -0.0002 0.0074 0.0016 0.0058 0.0147 0.7747 0.0000
